# Rapid emotional response and disadvantageous Iowa gambling task performance in women with borderline personality disorder

**DOI:** 10.1186/s40479-018-0092-x

**Published:** 2018-09-16

**Authors:** Jeannette LeGris

**Affiliations:** 1 Faculty of Health Sciences, School of Nursing, McMaster University, Ontario, Hamilton Canada; 2 Deparment of Psychiatry and Behavioural Neuroscience, McMaster University, Ontario, Hamilton Canada

**Keywords:** Iowa gambling task decision making, Borderline personality disorder, Impulsivity, Inhibitory control, Emotive impulsivity, Attention deficit scale for adults, ADHD co-morbidity, Affective dysregulation

## Abstract

**Background:**

Adults with Borderline Personality Disorder (BPD) manifest poor performance on tasks of decision making which may be congruent with their decisional and interpersonal conflicts in real life. Poor decision making is often assumed to be due to impulsive behaviour or weak inhibitory control despite inconsistent evidences of these relationships, leaving questions about the specific nature of these decisional deficits. Decision making in BPD may be compromised by different domains of impulsivity, affective dysregulatory processes or unknown co-morbid ADHD which is considered a developmental precursor to BPD.

**Findings:**

Iowa Gambling Task (IGT) decision making, 2 tasks of inhibitory control and a self report of ADHD symptoms consisting of 9 subscales were administered to 41 BPD women and 41 healthy controls. No group differences in inhibitory control were present. Net decision making performance and all ADHD subscale ratings differed significantly among BPD women and healthy controls. BPD women did not meet the threshold indicative of moderate to severe ADHD. Three subscales of attention, behaviour/ disorganized and emotive were significantly associated with poor IGT performance in 26 women with BPD. Of these 3 variables, the emotive subscale, representing a rapid emotional response, was the only significant predictor contributing 49% to the variance in poor DM.

**Conclusions:**

This is the 1st evidence of an emotive type of impulsivity, representing a type of affective instability that is linked to poor IGT DM in BPD. Findings support the Somatic Marker Hypothesis of IGT DM and may reflect the affective dysregulation that characterizes the disorder.

**Electronic supplementary material:**

The online version of this article (10.1186/s40479-018-0092-x) contains supplementary material, which is available to authorized users.

## Background

Many individuals with Borderline Personality Disorder (BPD) manifest poor decisions and risky impulsive behaviours in their daily lives and do not appear to readily learn from their prior experiences. The decisional conflicts of those with BPD may be congruent with their disadvantageous choices on decision making performance tasks [1, 2]. Of the 30 or more DM studies undertaken in BPD, few have reported normal performance [3]. Despite the trend of poor decisional performance in BPD, the precise nature of these decisional deficits remain unclear amid growing neuro-imaging evidence of fronto-limbic irregularities and dysfunctions which are proposed to underlie negative choice behaviour. Despite weak associations among lab measures of disadvantageous decision making (DM) and self reports of behavioural impulsivity [2, 4–7], assumptions of DM deficits are frequently attributed to impulsive behaviour or weak inhibitory control. DM represents complex impulsive behaviours consisting of emotional, cognitive and motivational dimensions [4, 8]. Understanding the unique dimensions of impulsivity may explain the poor DM performance of BPD subjects.

Most BPD symptoms are believed to arise from the core deficits of impulsivity and affective dysregulation however BPD theorists differ in their perspectives of which of these two complex phenomena constitute the primary deficit. Some favour impulsive behavior or impulse control deficits as key components of the disorder while others support the role of emotion dysregulation (ED) as the primary route to BPD. The ED route is proposed to result in impulsive behaviour, described as positive or negative urgency, which represent rash attempts to deal with intense positive or negative emotions [9]. The ED conceptualization suggests an interdependence between these core deficits where impulsive behaviour may depend more on emotional state than on impulsive personality traits or inhibitory control abilities. Emotional dysregulation and impulsive traits have been positively correlated in women with BPD [3, 8], however Fossati et al. [5] suggests that emotional dyregulation and difficulties with relationships precede deficits of impulse control and are dissociable from each other and other domains of impulsivity in BPD. A decisional or motivational facet of impulsivity is also reported to be more affected in BPD subjects than the purely cognitive components governing impulse control [2, 10, 11]. Disadvantageous decisional performance in BPD may occur in the context of strong positive or negative emotions indicative of an emotion dependent preference for immediate outcome irrespective of the magnitude of gain or loss.

Co-morbid ADHD in 19 to 38% of adults with BPD [10] is also proposed to explain even greater impulsive behaviour and motor control deficits in a subset of BPD subjects [10, 12]. ADHD is a disorder of inattention, hyperactivity and impulsivity with the recent inclusion of affective instability/dysregulation, as another primary factor [13] creating additional symptom overlap among these two disorders [14–17]. Interestingly ED and impulsivity were reported as unique predictors of BPD and unique mediators of the relation between childhood ADHD and adult BPD symptoms in women [5] raising questions about whether these disorders are distinct or two different expressions of the same syndrome [12, 18]. Individuals with ADHD are consistently characterized by motor inhibitory deficits [19], unlike BPD subjects who demonstrate intact motor control [2, 11, 20], implicating some specificity among these disorders. Individuals with BPD and co-morbid ADHD scored higher in all domains of impulsivity as assessed by the Barratt Impulsivity Scale relative to BPD subjects without ADHD co-morbidity [11]. Conversely, ADHD subjects with co-morbid BPD did not demonstrate higher impulsive ratings than those with ADHD alone, implicating differences in impulse severity or the type of impulsivity that characterizes each disorder. Thus there is a need to more fully understand the facets of ADHD impulsivity that may overlap with BPD symptoms and compromise decisional performance. As evidence begins to clarify these critical causal interrelationships, BPD researchers are proposing that both impulsive control and emotional regulation although related and overlapping may also represent distinct processes and behaviours which await further study and clarification.

The current report is a secondary analysis of original data from the first published study of Iowa Gambling Task (IGT, 19) performance and impulsivity as measured by the Attention Deficit Scale for Adults (ADSA, 20) in BPD women and Healthy Controls [2] where net IGT performance was unrelated to total ADHD symptom endorsements. The present analysis extends this work [2] by examining the unique facets/subscales of ADHD related impulsivity that may be more sensitive to disadvantageous IGT DM in BPD women. Specifically, this exploratory analysis will clarify i) the extent and type of adult ADHD symptom endorsement in BPD women relative to healthy controls and ii) will identify the unique facets of ADHD impulsivity and inhibitory control that best predict their poor IGT decisional performance. It was anticipated that i) ADHD co-morbidity would worsen disadvantageous IGT performance in BPD women and ii) the emotive and interpersonal impulsivity ADSA subscales would best predict disadvantageous IGT decision making as theoretically congruent with the affective processes of the IGT.

## Methods

### Participants

Data was collected during a prior study of cognitive function and IGT decision making in 42 out-patient women with BPD and 41 female healthy controls aged 18–50 years [2]. Participants with a confirmed BPD diagnosis and a current endorsement of 6 or more on the McLean Screen (MSI-BPD) [21] were eligible. BPD diagnosis was determined by DSM IV-TR criteria as assessed by the Structured Clinical Interview for DSM Axis II and the International Personality Disorder Examination conducted by trained PhD clinicians and a certified psychiatrist. Healthy Controls were staff and students from 2 teaching hospitals with no psychiatric illness who did not exceed a score of 2 on the MSI-BPD. Diagnostic interviews of DSM -IV criteria for co-morbid adult ADHD were not undertaken.

### Measures

The 54 *item Attention Deficit Scale for Adults (ADSA,* [22] contains 9 subscales representing different domains of ADHD pathology which correlate favourably with the DSM - IV criteria for ADHD. The ADSA is a reliable screen for adult ADHD which has been validated with BPD and ADHD subjects [23]Forty five of the 54 items relate to 6 different facets of ADHD impulsivity which include attention/concentration, behaviour/disorganized, interpersonal, emotive, consistency/long term, and negative social. This scale is more reliable among females than males [24], is one of the few scales that has been norm tested on clinical and non clinical populations and can discriminate among adults with and without ADHD. ADSA Items are scored on a 5 point likert scale with higher scores reflecting greater ADHD symptoms. The ADSA demonstrates an 88% sensitivity for diagnosing adults with ADHD on the basis of 4 subscales of inattention, behaviour disorganized, consistency/long term and negative social. A total ADSA score of > 181 representing a T score of .70 is indicative of highly likely ADHD. Prior research suggests that the consistency/long term subscale is the most discriminating type of impulsivity that distinguishes ADHD adults from BPD controls [25]. To date no research was located in which the ADSA was compared to the Barratt Impulsivity Scale nor the UPPS, two impulsivity scales which are frequently utilized in BPD research. Total ADSA and ADSA subscale scores were utilized in the current analysis.

The *Iowa Gambling Task (IGT)* is a sensitive, well established, reward processing decision probe highlighting the affective component of DM which mimics the uncertainty of real life. This task was initially developed to assess the decisional deficits of VMPFC lesioned patients and has subsequently been used extensively with many clinical populations [26, 27]. Performance on this task represents an ability to delay immediate reward in favour of longer term gain and requires self monitoring and implicit, intuitive learning from prior card selections. The ABCD computerized version of the IGT was used involving 100 card selections from 4 decks which are characterized by higher and lower magnitudes of punishment and different frequencies of punishment which together augment the ambiguity and complexity of one’s selections. IGT net scores of < 10 represent impairment [26] as no VMPFC patient to date has attained a net IGT score of 10. The IGT is proposed to be independent of education and IQ supporting its extensive use in clinical research.

As sex related differences in IGT performance have been frequently reported [27–29] with males outperforming females on this task, this sample was restricted to females. Damasio’s Somatic Marker Hypothesis [30] has guided the interpretation of IGT performance by proposing that deficits in emotional signaling result in poor social and interpersonal judgements. Emotions, indexed by autonomic bodily somatic markers, are proposed to mediate one’s reasoning at the point of deliberation, rather than as a consequence of the decision, which improves one’s decisions.

### Inhibitory control tasks

Two reaction time measures of inhibitory control assessed the ability to intentionally withhold or suppress attention or behavioural response to conflicting stimuli. One measure *(Stop Task),* [31] assessed behavioural inhibition of a prepotent response where longer stop signal reaction time (SSRT) represents weaker motor inhibitory control [32]. *The Victoria Stroop Task (VST)* [33] assessed interference control which tests the ability to selectively attend to conflicting tasks and repress automatic, interfering thoughts.

## Results

Data analyses were undertaken with SPSS version 24. One BPD subject had missing IGT data due to technical error.

Groups were similar in age, IQ, and marital and Canadian born status but differed significantly on full time employment or student status and years of education although the BPD group was considered well educated (M = 13.5 yrs., SD 10.5). 50% of the clinical group endorsed prior substance abuse/dependence. 85% of the clinical group experienced 2 co-morbid disorders most frequently involving anxiety. Childhood ADHD was suspected by 3 BPD subjects but without formal diagnosis. Only 1 BPD subject endorsed childhood ADHD. 76% of the BPD sample was stabilized on prescribed psychotropics. All subjects were free of recreational drug use at time of testing [2]. Due to the exploratory nature of this study, bonferonni corrections for multiple comparisons were not applied.

Sixty three percent (26/41) of the BPD sample and 15/41 (36%) of controls had net decision scores < 10, indicative of below average performance. Group differences in net decision making (d = 0.72) in BPD subjects (M = −.71 SD 30.2) and healthy controls (M = 21.07 SD 30.3) remained significant despite control for IQ, education, psychotropic use, depression, anxiety, and substance abuse history [2]. Mean total ADSA and all ADSA subscale scores differed significantly among BPD women and controls (Table 1). Mean total ADSA scores in BPD subjects (M = 177.6, SD 20.1) did not meet the threshold of moderate to severe ADHD (M > 181, [22] however 17 of 42 BPD women exceeded total ADSA scores of 181. To compare differences in net IGT performance among BPD subjects, total ADSA scores were dichotomized into high (182–212, *n* = 10) and low categories (123–181, *n* = 16). There were no significant differences in net IGT performance among BPD women with high (M, − 12.4, SD, 19) or low ADSA ratings (M = − 21, SD,26, *p* = 0.62). Counterintuitively, those with greater IGT impairment were categorized in the low ADSA category. A chi square analysis of group differences between good and poor IGT (< 10) performance and high and low ADSA(< 181) ratings in BPD women and healthy controls also demonstrates a non significant relationship (x^2^ = 1.14, df 1, *p* = 0.29). To confirm whether greater ADHD co-morbidity was associated with IGT decision making in the total sample, a logistic regression analysis of advantageous and disadvantageous IGT performers (net IGT < 10) as the dependent variable with the predictors of high and low ADSA endorsements and Group status (BPD or HC) was computed. Findings revealed that group was the sole predictor of IGT decision making (Group: B = 1.08, SE .50 df 1, Exp (B) 2.96, *p* = 0.03. High and low ADSA summary scores were not significant to IGT performance (B = −.05 SE .61, df1, Exp(B) = .29, *p* = 0.94), thus disproving hypothesis 1.

**Table 1:** Mean ADSA Total and Subscale Scores

*ADSA Scale*	*Women with BPD*	*Non Clinical Controls*	*p value (df 81)*
	*M (SD)*	*M (SD)*	
*Attention/Concentration*	*42.6 (7.2)*	*32.9 (6.2)*	*.000*
*Interpersonal*	*26.8 (3.4)*	*19.7 (3.0)*	*.000*
*Behaviour/Disorganized*	*76.9 (9.3)*	*59.0 (12.4)*	*.000*
*Coordination*	*8.7 (3.2)*	*5.9 (2.0)*	*.000*
*Academic*	*6.5 (1.4)*	*5.5(1.5)*	*.002*
*Emotive*	*35.3 (5.6)*	*24.1 (4.9)*	*.000*
*Consistency/Long Term*	*37.5 (4.8)*	*29.4 (4.1)*	*.000*
*Childhood*	*5.8 (2.0)*	*5.0 (1.4)*	*.03*
*Negative/Social*	*19.3 (3.6)*	*14.3 (3.0)*	*.000*
*ADSA Total*	*177.8 (20.1)*	*135.3 (20.33)*	*.000*

**Fig. 1:**
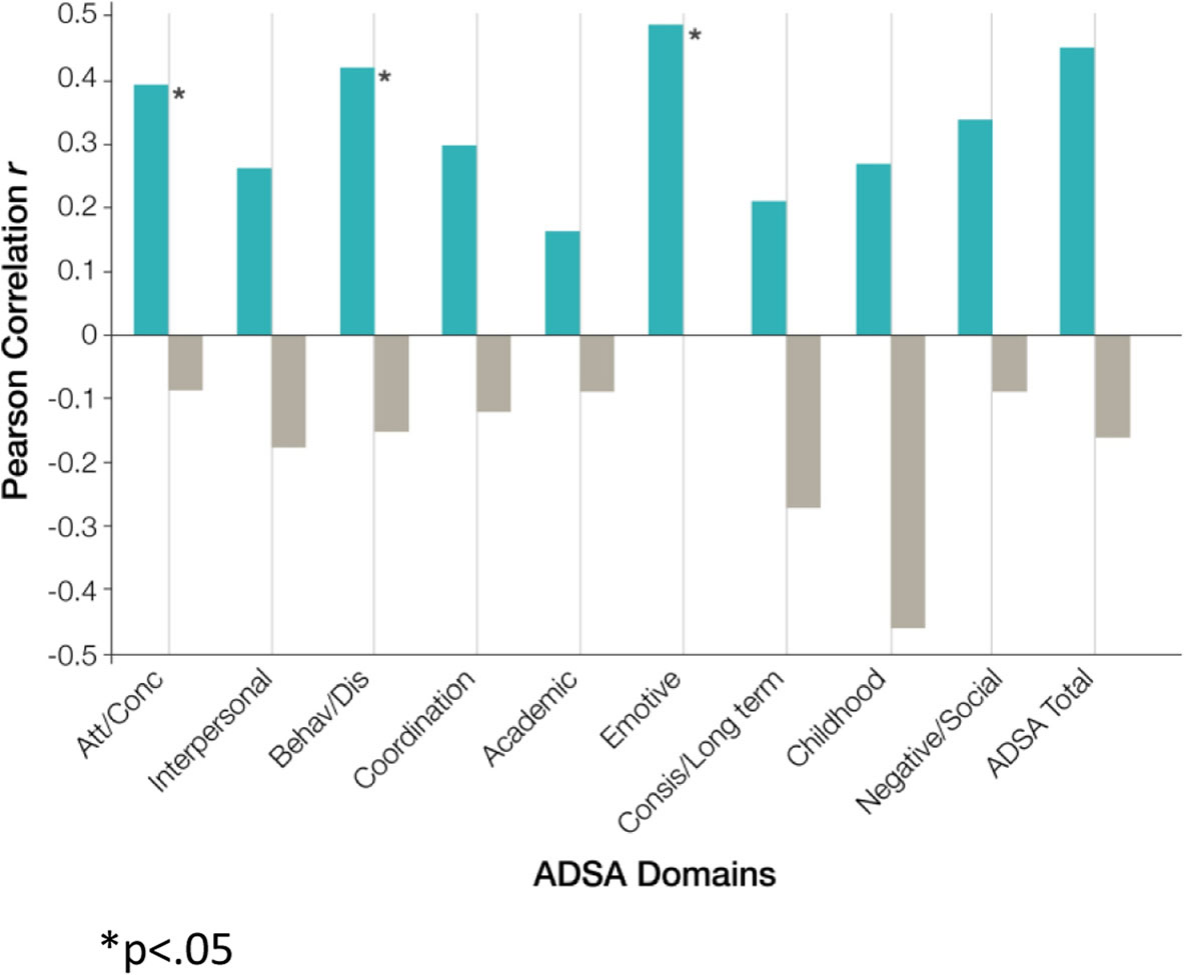
ADSA Correlates and poor IGT Performance

Pearson product correlations of total ADSA and all ADSA subscale scores with *poor IGT DM* in 26 BPD women and 15 HC are depicted in Fig. 1. Total ADSA scores were significantly associated (*r* = .45, *p* = 0.01) with *poor IGT (< 10)* performance in BPD subjects only, but were not significant to their overall net IGT performance (*r* = .23, *p* = 0.14 *n* = 41). Weak associations among total ADSA scores and *poor IGT* performance in the total sample were also evident (*r* = .04, *p* = 0.81, *n* = 82). ADSA Total scores and *net IGT* performance were also non significant in the total sample (*r* = −.21, *p* = 0.06, n = 82). Only 3 of 9 ADSA subscales were significantly associated with poor IGT DM in BPD women: Emotive,(*r* = .49, *p* = 0.01); Behaviour/Disorganized (*r* = .42,*p* = 0.04); and Attention/concentration (*r* = .39, *p* = 0.05). Neither measure of inhibitory control was significantly associated with poor DM in BPD women (Table 2).

**Table 2:** Inhibitory Control, Impulsivity and Poor IGT Performance

*Variables*	*Women with BPD*
	*n = 26*
	*r*
*Cognitive Control (Stroop)*	*−.37*
*Motor Control (SSRT)*	*.22*
*Impulsivity (ADSA total)*	.45*

Poor IGT Performance = net IGT score < 10

*p < .05

A second logistic regression analysis (Table 3) predicting group status with the 6 ADSA impulsivity subscales as predictors revealed that only the emotive and interpersonal subscales remained significant correctly classifying 93% of BPD women and 90% of healthy controls (x^2^ = 80.31, df1 *p* = .000 *n* = 83). A final analysis of *poor IGT performance in women with BPD*, involving emotive, behaviour/disorganized, attention/concentration and interpersonal predictors were entered into a multiple regression model to address hypothesis 2 (Table 4). Although not statistically significant to poor IGT DM, interpersonal was included, due to its clinical and theoretical relevance to IGT performance representing interpersonal conflict. Collectively these 4 variables explained 21% of the adjusted variance in poor IGT DM [F (1,24) = 7.66 *p* = 0.01] however only the emotive subscale remained significant (β = 0.49, *p* = 0.01) in partial support of hypothesis 2. These results remained stable when these same predictor variables were entered via forward, backward or simultaneous methods. To examine whether current depression or anxiety contributed additionally to the emotive prediction of poor DM; BAI, BDI ratings and the emotive variable were entered simultaneously into a 2nd multiple regression analysis. Emotive remained the sole predictor of poor IGT performance with BAI and BDI dropping out of the model.

**Table 3:** Logistic Regression Predicting Group Status using 6 ADSA Impulsivity Subscales (*n* = 83)

Variable	B	SE	df	Exp B	Significance
Attent/Conc	.26	.14	1	1.3	.07
Behav/Disorg	.05	.09	1	1.1	.60
Emotive	−.50	.22	1	.61	.02^*^
Interpersonal	−.59	.27	1	.56	.03^*^
Consistency/LT	−.30	.19	1	.74	.12
Neg.Social	−.02	.18	1	1.0	.92

* = *p*<.05

x^2^ = 80.31, df 1, *p* = 0.00 93% of BPD and 90% of Healthy Controls correctly classified

**Table 4:** Impulsivity Domains and Poor IGT Decision Making in Women with BPD

*ADSA subscales*	*Model 1*			*Model 2*			*Model 3*			*Model 4*		
	*B*	*SE*	ß	*B*	*SE*	P	*B*	*SE*	ß	*B*	*SE*	ß
*Attention/Concentration*	*.00*	*.98*	*.00*									
*Interpersonal*	*.35*	*1.38*	*.05*	*.35*	*1.35*	*.05*						
*Behaviour/Disorganized*	*.30*	*.91*	*.10*	*.30*	*.84*	*.10*	*.30*	*.82*	*.10*			
*Emotive*	*1.60*	*1.21*	*.39*	*1.60*	*1.21*	*.39*	*1.70*	*1.13*	*.42*	*2.00*	*.72*	.49*
*Constant*	*− 107.0*	*49.54*		*−107.0*	*48.29*		*− 101.4*	*42.14*		*−89.42 26.04*		

F = 7.66 *p = .01; *r*^*!*^ = .24, adj r^3^ = .21

Additional data analysis depicting the predictors of group status is available in (Additional file 1: Table S5) and (Additional file 2: Table S6). All logistic regression models (Table 2, Additional file 1: Table S5 and Table S6) demonstrated non significant Hosmer and Lemeshow Tests and Cox and Snell R2 and Nagelkerke R2 estimates.

## Discussion

ADHD symptom endorsement in recently treated BPD subjects and the associations of ADHD related symptoms and impulsivities with poor IGT performance were examined. While BPD women differed significantly from healthy controls on all ADSA subscale ratings, their mean total ADSA endorsements did not meet the threshold for ADHD. Mean ADSA scores in adults with ADHD were reported to be higher (M = 186, SD 25) [22] than the mean scores of the current BPD sample. As the primary discriminatory symptoms of ADHD (attention, behav/disorg, consistency/LT and negative social) were not predictive of BPD status, ADHD co-morbidity in the present BPD sample appears unlikely. Total ADSA scores were unrelated to net IGT DM performance in the BPD and the total sample [2] in contrast to the significant associations of total ADSA ratings in BPD women performing *poorly* on the IGT. Three of 6 ADSA impulsivity subscales (attention, behav/disorg and emotive) were moderately associated with poor IGT performance in BPD subjects, however only the emotive subscale, representing a rapid emotional response, predicted their below average decisional performance. ADHD symptom endorsement in BPD women appears to be primarily due an emotive type of impulsivity involving rapid shifts in mood, which may compromise their attention, reward processing abilities and goal seeking behaviour. Present findings may be congruent with reports of greater affective reactivity [17, 34] and normal inhibitory control in BPD adults relative to ADHD adults [11]. In contrast to the predominant motor inhibitory deficits which tend to characterize ADHD adults, BPD women’s cognitive and motor inhibitory control was unrelated to their poor IGT performance. This is the first evidence of an association between an emotive type of impulsivity and poor IGT performance which may be congruent the role of emotional dysregulation and negative urgency experienced by BPD women during IGT performance [30, 35]. Some items on the emotive subscale include “ I tend to overreact, good and bad moods are easily triggered, I feel overwhelmed by all the things I need to do, I feel stressed by the demands of others, I get agitated quickly, I am easily excitable and I do not have patience with difficult tasks”. These items may reflect characteristics attributed to negative urgency, where rash decisions are made under contexts of negative affect. This is the 2nd known study to use the ADSA with BPD participants. Mean ADSA subscale scores in the present BPD sample were remarkably similar to those reported by Dowson [25] who found relationships between attention, behav/disorg and emotive and the Tower of London (TOL) planning task but were unrelated to the Cambridge Gamble Task of *unambiguous* decision making. Although speculative, the TOL may represent processes of future oriented thinking and uncertainty, not unlike the *ambiguous* IGT.

Some specificity for BPD impulsivity relative to adult ADHD impulsivity is implicated, however an ADHD control group is needed to confirm present results. Future research should include multiple measures of impulsivity which capture a range of dimensions beyond the behavioural/cognitive traits. A preference for total scores versus subscale analyses in prior research may have resulted in the weak associations of self reported impulsivity and the lab tasks of DM as previously reported. Despite the complexities, conceptual ambiguities and the multi-dimensional nature of DM, impulsivity and affect dysregulation, this line of inquiry may eventually lead to novel assessments and treatments targeting the unique mechanisms underlying ambiguous decision making behaviours. As more refined tasks of affect and affect dysregulation emerge, the role of affect and impulsivity or affect versus impulsivity in the development of BPD may eventually be clarified. Findings may have implications for a broader understanding of the emotional components of DM which may precede risky behaviour in BPD, as suggested by others [5, 10] Present findings support Sebastien’s premise that impulsivity in BPD, if not caused by ADHD, may be another sequel of emotional dysregulation [10]. Although cross sectional findings cannot infer causal relationships, examining the associations of impulsive behaviour, affect and DM among children and adolescents with BPD like symptoms in future studies may clarify the direction of these potential developmental precursors to BPD. Current findings support the notion that IGT decision making appears to be more dependent upon emotional reactivity or instability rather than depressive or anxious state and other types of impulsive behaviour in women with BPD. This decisional impairment may represent the interpersonal traits of BPD which are slower to remit [36] and the debilitating psychosocial impairment that tends to persist beyond the diagnostic threshold for the disorder.

## Additional files

Additional file 1: **Table S5.** Logistic regression predicting Group Status on the basis of Total ADSA scores and net IGT performance in 41 BPD women and 41 healthy controls. (PDF 85 kb)

Additional file 2: **Table S6.** Logistic regression predicting Group status on the basis of net IGT performance and 3 ADSA subscales in 41 BPD women and 41 Healthy Controls. (PDF 98 kb)
