# Supplementary material for: Rapid emotional response and disadvantageous Iowa gambling task performance in women with borderline personality disorder
Source: Borderline Personal Disord Emot Dysregul. 2018 Sep 16;5:16. doi: 10.1186/s40479-018-0092-x (PMC6139153; doi:10.1186/s40479-018-0092-x)
Supplement: Supplementary file 1 — Table S5. Logistic regression predicting Group Status on the basis of Total ADSA scores and net IGT performance in 41 BPD women and 41 healthy controls. (PDF 85 kb) [file 40479_2018_92_MOESM1_ESM.pdf]

Additional File 1

Table 5 Logistic Regression Predicting Group Status on the basis of Total ADSA scores and net IGT Performance

Variable	B	SE	Odds Ratio	P value
Net IGT DM	.03	.01	1.03	.02
ADSA Total	-.09	.02	.91	.000
Constant	13.98	2.97	1179207.13	

90.2 % of Healthy Controls and 90.2 % of BPD subjects were correctly classified (X2 = 61.94, df=2, n= 82. p = .000)

Table 6. Logistic Regression Predicting Group Status on the basis of net IGT Performance and 3 ADSA Subscales

Variable	B	SE	Odds Ratio	P value
Net IGT	.03	.01	1.03	.03
Attent/Conc	.09	.10	1.10	.34
Behav/Disorg	-.05	.07	.95	.51
Emotive	-.42	.14	.66	.003
Constant	12.03	3.17	167631.68	

This model correctly classifies 90.2 % of BPD women and 85% of healthy controls (X2 = 65.58, df= 4, n = 82, p = .000)

All logistic regression models (Tables 3, 5,6) demonstrate non significant Hosmer and Lemeshow Tests and Cox amd Snell R2 and Nagelkerke R2 estimates.
